# The economic impact associated with stent retriever selection for the treatment of acute ischemic stroke: a cost–effectiveness analysis of MASTRO I data from a Chinese healthcare system perspective

**DOI:** 10.57264/cer-2024-0160

**Published:** 2024-11-05

**Authors:** Osama O Zaidat, Xinguang Yang, Waleed Brinjikji, Emilie Kottenmeier, Hendramoorthy Maheswaran, Thibaut Galvain, Patrick A Brouwer, Mahmood Mirza, Tommy Andersson

**Affiliations:** 1 Mercy St Vincent Medical Center, Toledo, OH 43608, USA; 2 Sun Yat-sen Memorial Hospital, Sun Yat-sen University, Guangzhou, Guangdong Province, 510123, China; 3 Department of Radiology, Mayo Clinic, Rochester, MN 55902, USA; 4 Cardiovascular & Specialty Solutions Group, CERENOVUS, Irvine, CA 92618, USA; 5 Johnson & Johnson MedTech, Global Health Economics, New Brunswick, NJ 08901, USA; 6 Medical Imaging, AZ Groeninge, 8500, Kortrijk, Belgium; 7 Neuroradiology, Karolinska University Hospital & Clinical Neuroscience Karolinska Institute, 171 77, Stockholm, Sweden

**Keywords:** acute ischemic stroke, EmboTrap, functional outcomes, MASTRO I, mechanical thrombectomy, modified Rankin Scale, Solitaire, stent retriever, Trevo

## Abstract

**Aim:**

The aim of this analysis was to assess the cost–effectiveness of the EmboTrap^®^ Revascularization Device compared with the Solitaire™ Revascularization Device and Trevo^®^ Retriever for the treatment of acute ischemic stroke (AIS) from the perspective of the Chinese healthcare system.

**Methods:**

According to MASTRO I, a recent living systematic literature review and meta-analysis, mechanical thrombectomy (MT) with EmboTrap in the treatment of AIS resulted in better functional outcomes compared with the use of Solitaire or Trevo. Based on the proportion of patients that achieved 90-day modified Rankin Scale (mRS) scores of 0–2, 3–5 and 6 reported in MASTRO I, a combined 90-day short-term decision tree and Markov model with a 10-year time horizon was used to compare the cost–effectiveness of the three devices. The primary outcome was the incremental cost–effectiveness ratio (ICER), representing the incremental cost (in 2022 Chinese Yuan [CNY]) per incremental quality-adjusted life-year (QALY). The ICERs were compared against willingness-to-pay (WTP) thresholds of 1, 1.5 and 3-times the 2022 national gross domestic product (GDP) per capita in China.

**Results:**

Treatment with EmboTrap resulted in total QALYs of 3.28 and total costs of 110,058 CNY per patient. Treatment with Trevo resulted in total QALYs of 3.05 and total costs of 116,941 CNY per patient. Treatment with Solitaire resulted in total QALYs of 2.81 and total costs of 99,090 CNY per patient. Trevo was dominated by EmboTrap as it was a more costly and less effective intervention. As such, Trevo was not cost-effective at any WTP threshold. Compared with Solitaire, EmboTrap was more effective and more costly, with an ICER of 23,615 CNY per QALY. This result suggests that EmboTrap is cost-effective when compared with Solitaire since the ICER was lower than all WTP thresholds assessed.

**Conclusion:**

EmboTrap dominated Trevo and is cost-effective for the treatment of patients with AIS compared with Solitaire when assessed from the perspective of the Chinese healthcare system and based on the device-level meta-analysis MASTRO I. Selecting a stent retriever (SR) that optimizes 90-day mRS score is an important consideration from both a clinical and healthcare payer perspective in China as it is associated with reduced long-term costs and increased quality of life.

## Plain language summary

**What is this article about?:**

Mechanical thrombectomy (MT) using stent retrievers (SRs) is strongly recommended by the Chinese Stroke Association for the treatment of acute ischemic stroke (AIS) in eligible patients in China. MASTRO I, a recently published systematic review and meta-analysis, compared outcomes following MT for three commonly used SRs (EmboTrap^®^, Solitaire™ and Trevo^®^) and found that the SR used during MT may impact functional outcomes. Data from MASTRO I indicated that compared with Solitaire and Trevo, MT with EmboTrap was associated with improved functional outcomes. As better functional outcomes may result in lower healthcare resource utilization, SR choice during MT may have downstream economic implications. Therefore, the aim of the current analysis was to compare the cost–effectiveness of EmboTrap, Solitaire and Trevo from the perspective of the Chinese healthcare system over a 10-year time horizon based on functional outcomes 90-day post-stroke reported in MASTRO I.

**What were the results?:**

EmboTrap dominated Trevo and was cost-effective compared with Solitaire when assessed at willingness-to-pay (WTP) thresholds of 1, 1.5 and 3-times the 2022 China national gross domestic product (GDP) per capita.

**What do the results mean?:**

The results of this analysis indicate that EmboTrap may offer better value for money compared with Solitaire and Trevo in the treatment of AIS and that the downstream economic consequences of SR selection during MT are an important consideration from a clinical and healthcare payer perspective.

## Graphical abstract

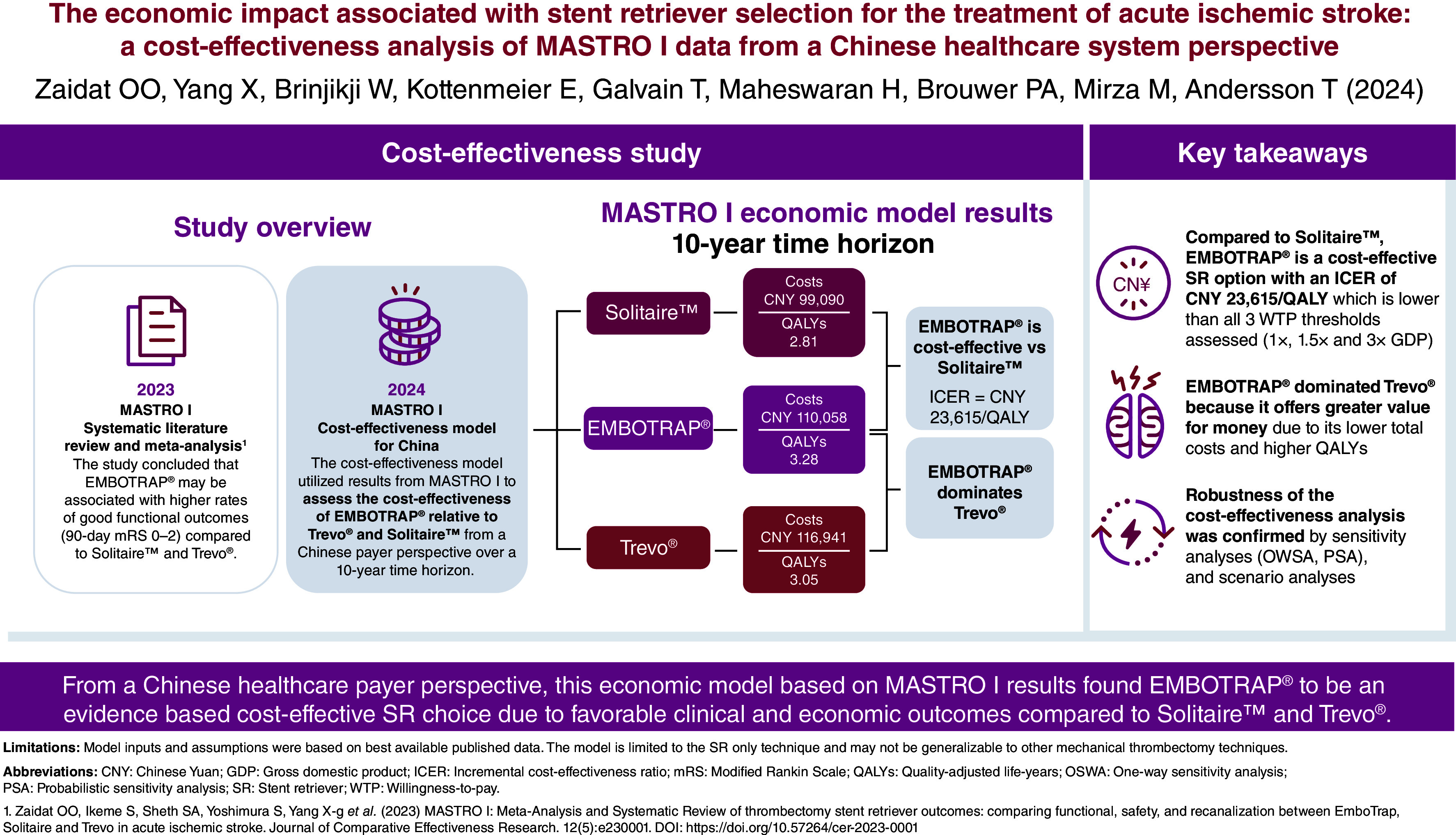

Globally, 87% of strokes are ischemic, 10% are intracerebral hemorrhage and 3% are subarachnoid hemorrhage [1]. Aligning with global data, ischemic stroke is the most common form of stroke in China [2,3], accounting for 86.8% of all incident strokes in 2020 [3]. Approximately 35–40% of all acute ischemic strokes (AIS) are due to proximal large vessel occlusions (LVO) [4,5]. According to the 2019 update on management of ischemic cerebrovascular diseases released by the Chinese Stroke Association, mechanical thrombectomy (MT) is strongly recommended for eligible patients with AIS and should be performed as the first-line treatment for patients who are ineligible for intravenous recombinant tissue plasminogen activator (IV-tPA) [6]. Patients who are eligible for IV-tPA should receive a combination therapy of IV-tPA and MT [6]. When performing MT, stent retrievers (SRs) are recommended as the first choice of thrombectomy device; however, the operator may choose to use other thrombectomy or aspiration devices at their own discretion (subject to SR approval by regional health authorities) [6]. Although other devices can be used during MT, SRs are proficient at achieving successful reperfusion as a stand-alone first-line technique [7]. While all SRs share the common goal of removing the occlusion and achieving reperfusion, SRs have distinct design features and mechanisms of action that may impact clinical outcomes [8–11].

The results of the recent MASTRO I living systematic literature review and meta-analysis suggest that SR choice may impact clinical outcomes following MT [9]. Using the Solitaire™ Revascularization Device during MT resulted in significantly higher rates of symptomatic intracranial hemorrhage (sICH; 7.7%) compared with the EmboTrap^®^ Revascularization Device (3.9%, p = 0.028) and the Trevo^®^ Retriever (4.6%, p = 0.049); however, there was no significant difference between EmboTrap and Trevo (p = 0.514). Similarly, using Solitaire during MT resulted in significantly higher mortality rates (20.4%) compared with using EmboTrap (11.2%, p < 0.001) or Trevo (14.5%, p = 0.018); however, there was no significant difference between EmboTrap and Trevo (p = 0.127) [9]. Finally, MASTRO I demonstrated that the use of EmboTrap during MT resulted in significantly higher rates of good functional outcomes (57.4%; defined by modified Rankin Scale [mRS] score of 0–2 at 90 days post-stroke) compared with the use of Solitaire (45.3%, p < 0.001) or Trevo (50.0%, p = 0.013) [9].

An inverse relationship exists between mRS score and patient quality of life (QoL) [12]. Furthermore, good functional outcomes (i.e., low mRS score) post-stroke may reduce the economic burden of stroke since medical costs increase with rising 90-day post-stroke mRS score [13,14]. This highlights the potential cost savings associated with achieving good functional outcomes following MT. While a previous study found that endovascular treatment combined with standard medical treatment is cost-effective compared with standard medical treatment alone in Chinese patients with basilar artery occlusion, the study did not assess the economic impact of endovascular device selection [15]. Given the economic burden of stroke in China and the paucity of cost–effectiveness studies directly comparing SRs, it is important to understand the economic impact of SR selection, particularly in relation to functional outcomes following MT. While device selection is influenced by surgeon preference, institutional factors and patient vascular and thrombotic conditions, understanding its impact on cost can be beneficial when multiple SR devices are viable options. The aim of the current study was to address this gap by evaluating the cost–effectiveness of three commonly used SRs (EmboTrap, Solitaire and Trevo) based on the proportion of patients with AIS who achieved good functional outcomes following MT as reported in MASTRO I.

## Methods

### Model overview

**Figure 1.**
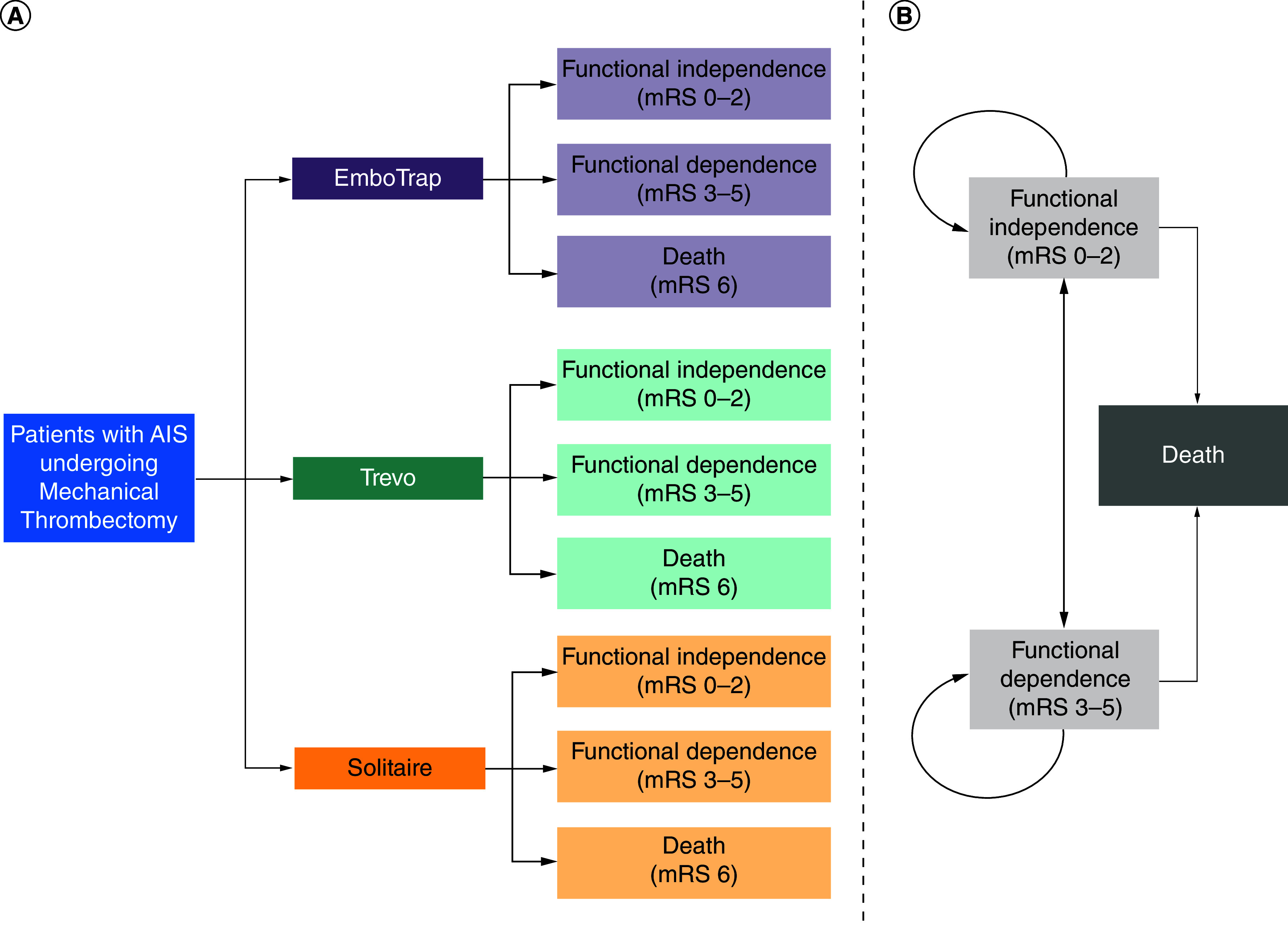
Cost–effectiveness analysis model structure. **(A)** A combined short-term decision tree and **(B)** long-term Markov model. The decision tree spanned 90 days and the Markov model had cycles lasting 1 month. The total Markov simulation period was 10 years. In the long-term Markov model, patients could transition between functional dependence and functional independence during the first year following stroke. After the initial year, patients who achieved functional independence could transition to functional dependence or death up to 5 years post-stroke, after which they could only transition to death. After the first year following stroke, patients with functional dependence could only transition to death. AIS: Acute ischemic stroke; mRS: Modified Rankin Scale.

An analysis was conducted to assess the cost–effectiveness of EmboTrap versus Trevo versus Solitaire for the treatment of eligible patients with AIS (Figure 1). The analysis assessed the cost–effectiveness of the SRs from the perspective of the Chinese healthcare system and was designed to estimate the differences in costs and QALYs. Adherence to the 2020 China Guidelines for Pharmacoeconomic Evaluations was maintained when conducting the analyses and the Consolidated Health Economic Evaluation Reporting Standards (CHEERS) were followed for reporting [16,17]. The model was developed using R statistical software (R version 4.3.0) [18].

The population of interest was patients with AIS due to LVO treated with either EmboTrap, Solitaire, or Trevo as the first-line device in a stent-retriever only procedure. A recent study found that the median age of patients eligible for intravenous thrombolysis and endovascular therapy for AIS was 67 years of age [19]. In accordance with standard modeling practice, patient age was rounded to the nearest decade and the base case analysis assumed patients were 70 years of age. Functional outcomes reported in MASTRO I [9] were utilized in this analysis. For information related to study identification and data handling, please refer to the MASTRO I publication [9]. All-cause mortality was informed by Chinese life tables from the 2020 National Population Census [20].

### Model structure

The model was based on a previously published model [21] and combined a short-term (index hospitalization) decision tree and long-term (10-year) Markov cohort state-transition model that allowed for heterogeneous variations in transition probabilities over time (Figure 1). The Markov model had three mutually exclusive health states: functional independence (mRS 0–2), functional dependence (mRS 3–5), or death (mRS 6; an absorbing health state). The short-term decision tree simulated the outcomes after patients with AIS underwent MT with either EmboTrap, Solitaire, or Trevo. The proportion of patients in each health state was informed by MASTRO I, which provided pooled estimates for achieving functional independence (mRS 0–2), functional dependence (mRS 3–5), or death (mRS 6) at 90 days for the three SRs of interest [9]. Patients entered the corresponding health state based on the reported 90-day mRS in the Markov model.

In the Markov model, patients could transition between ‘functional dependence’ and ‘functional independence’ only during the first-year post-stroke. After the first year, patients in the ‘functional dependence’ health state could only transition to ‘death’. Relatedly, after the first-year post-stroke, patients in the ‘functional independence’ health state could transition to the ‘functional dependence’ or ‘death’ states for four additional years (i.e., a total of 5 years post-stroke), after which they could only transition to ‘death’. In addition to stroke-related deaths, general population mortality was captured based on 2020 life table data from the National Bureau of Statistics of China [20] (Supplementary Table 1). It was assumed that recurrent stroke is accounted for in mRS transition probabilities.

### Transition probabilities

The proportion of patients in each health state (functional independence, functional dependence or death) in the decision tree were derived from MASTRO I [17] (Supplementary Table 1).

Data from a previous health technology assessment of MT from Canada [21] was used to model long-term transition probabilities between the three health states in the Markov model (Supplementary Table 1). The assessment used 5-year survival data from the Oxford Vascular Study [22,23] to estimate the proportion of patients with AIS who were functionally independent (mRS 0–2), functionally dependent (mRS 3–5) and dead (mRS 6) at different time points. The risk of mortality after 90 days for functionally independent and functionally dependent patients was calculated by multiplying the risk ratios (RR) reported in a previous health technology assessment of MT from Canada [21] by the age-specific all-cause mortality for the Chinese population [20] (Supplementary Table 1). A previously reported calibration process [24] was used to derive the required monthly transition probabilities that achieved a good fit to the observed survival data.

### Costs

All costs were considered from the perspective of the Chinese healthcare system and were inflated to 2022 Chinese Yuan (CNY) using data reported by the National Bureau of Statistics of China [25]. An annual discount rate of 5% was applied to costs in alignment with the China Guidelines for Pharmacoeconomic Evaluations [17].

The analysis included SR device cost, the cost of index hospitalization post-MT and the annual cost of long-term care (Supplementary Table 2). The costs of the SRs were sourced from the Taimao Medical Device Tendering Network [26]. The index hospitalization cost (after MT) for patients with mRS 0–2 and mRS 3–5 was sourced from a recently published real-world study that quantified healthcare resource use and costs among patients enrolled in the China National Stroke Registry-III (CNSR-III) who survived their first admission with AIS [27]. As the real-world study did not report a cost for patients who died, the index hospitalization cost for patients with mRS 6 was calculated by multiplying the index hospitalization cost estimated for patients with mRS 0–2 [28] by the ratio of index hospitalization costs (mRS 0–2 to mRS 6) reported in an economic evaluation of Chinese stroke patients [29]. The annual cost of long-term care for patients with mRS 0–2 or 3–5 was obtained from the same economic evaluation of Chinese stroke patients [29] (Supplementary Table 2).

### Utilities

Health utilities for the base case analysis were obtained from a study that mapped mRS scores and health-related QoL to the European Quality of Life 5-dimensional questionnaire (EQ-5D) to derive utility-weight stroke outcomes measures [30]. Individual participant data was pooled from eight randomized multicenter stroke trials consisting of over 20,000 patients (43.1% of which were Chinese) to calculate utility-weighted scores across mRS 0 to 5 [30]. Based on the proportion of patients classified as mRS 0–2 and 3–5 in MASTRO I, a weighted average of these scores was used to derive EQ-5D utility scores for functional independence (mRS 0–2) and functional dependence (mRS 3–5) (Supplementary Table 2). Quality-adjusted life years (QALYs) were calculated by multiplying corresponding utility scores by the number of months a patient spent in each health state. An annual discount rate of 5% was applied to QALYs in alignment with the China Guidelines for Pharmacoeconomic Evaluations [17].

### Primary outcomes

The costs and QALYs over a 10-year time horizon were estimated for a hypothetical cohort of 1000 patients with AIS that underwent MT using one of the three SRs. The primary outcome for the comparisons was the incremental cost–effectiveness ratio (ICER) representing the incremental cost (in CNY) per incremental QALY.

A SR was considered cost-effective if the resulting ICER was lower than the willingness-to-pay (WTP) threshold. In China, WTP thresholds informing reimbursement of health technologies are based on the national gross domestic product (GDP) per capita. While it has been previously suggested that ICERs below 1.5-times the GDP per capita are considered cost-effective [31], the 2020 China Guidelines for Pharmacoeconomic Evaluations have advised ICERs less than 1 to 3-times the national GDP per capita are considered cost-effective [17]. With a GDP per capita of 85,698 CNY in 2022 [25], the model results were compared against thresholds of 1, 1.5 and 3-times the GDP per capita (i.e., 85,698 CNY/QALY, 128,547 CNY/QALY, and 257,084 CNY/QALY, respectively).

### Sensitivity & scenario analysis

To determine which variables have the largest impact on results, deterministic one-way sensitivity analyses (OWSA; i.e., EmboTrap vs Solitaire [Supplementary Figure 1], Trevo vs EmboTrap [Supplementary Figure 2] and Trevo vs Solitaire [Supplementary Figure 3]) were performed in which input parameters were varied individually over the extremities of their 95% confidence interval (when available) or by ±10% of their base case value.

Scenario analyses were performed assessing alternative time horizons (1 year, 5 years and 15 years), a population starting age of 65 years and utilizing utility values from an alternative published source [29,32]. Additional hypothetical scenario analyses were conducted in which the device cost for Solitaire and Trevo were varied to determine the lowest hypothetical cost of these devices at which point EmboTrap is no longer estimated to be cost-effective at the most conservative WTP threshold of 85,698 CNY/QALY (1 times the GDP per capita).

Probabilistic sensitivity analyses (PSAs) were conducted by generating 1000 iterations of parametric Monte Carlo simulations to further assess the uncertainty of the results. Utilities were modeled using a beta distribution and costs were modeled using a gamma distribution [33]. Transition probabilities were modeled using a combination of multinominal, beta and log normal distributions [33]. Cost–effectiveness acceptability curves were generated to facilitate comparisons and to visualize the probability of the SRs being cost-effective at increasing WTP thresholds.

## Results

### Deterministic base case analysis

Over a 10-year model time horizon, EmboTrap was associated with 3.28 QALYs, and total costs of 110,058 CNY per patient. Treatment with Trevo afforded patients 3.05 QALYs and was associated with total costs of 116,941 CNY per patient. Patients treated with Solitaire accumulated 2.81 QALYs, and total costs of 99,090 CNY per patient. Compared with EmboTrap, Trevo was a more costly (116,941 CNY per patient vs 110,058 CNY per patient for EmboTrap) and less effective intervention (3.05 QALYs vs 3.28 QALYs for EmboTrap). As such, Trevo was dominated by EmboTrap and was not cost-effective at any WTP threshold.

In relation to Solitaire, the use of EmboTrap resulted in an ICER of 23,615 CNY/QALY and was considered cost-effective since the ICER was less than the per capita GDP in 2022 in China (85,698 CNY/QALY) (Table 1).

**Table 1.** Cost–effectiveness results from deterministic base case, deterministic scenario analyses, and probability of cost–effectiveness.

Device	Discounted mean costs (2022 CNY) and QALYs per person				ICER (CNY/QALY)^‡^	Probability cost-effective at cost–effectiveness threshold^¶^		
	Mean total cost (CNY)	Incremental cost (CNY)	Mean QALYs	Incremental QALYs		85,698 CNY/QALY	128,547 CNY/QALY	257,084 CNY/QALY
**Base case (age 70 years, 10-year time horizon, EQ-5D)**								
Solitaire	99,090	–	2.811	–	–	0.000	0.000	0.000
EmboTrap	110,058	10,968	3.276	0.464	23,615^§^	1.000	1.000	1.000
Trevo	116,941	–^†^	3.047	–^†^	Dominated by EmboTrap^#^	0.000	0.000	0.000
**Age 65 years**								
Solitaire	104,928	–	3.030	–	–	0.000	0.000	0.000
EmboTrap	116,335	11,408	3.520	0.489	23,318^§^	1.000	1.000	1.000
Trevo	123,166	–^†^	3.282	–^†^	Dominated by EmboTrap	0.000	0.000	0.000
**1-year time horizon**								
Solitaire	53,541	–	0.494	–	–	0.276	0.037	0.000
EmboTrap	59,353	5,812	0.582	0.088	66,267^§^	0.724	0.963	1.000
Trevo	68,037	–^†^	0.536	–^†^	Dominated by EmboTrap	0.000	0.000	0.000
**5-year time horizon**								
Solitaire	79,062	–	1.867	–	–	0.000	0.000	0.000
EmboTrap	87,620	8,557	2.179	0.311	27,481^§^	1.000	1.000	1.000
Trevo	95,410	–^†^	2.024	–^†^	Dominated by EmboTrap	0.000	0.000	0.000
**15-year time horizon**								
Solitaire	108,089	–	3.232	–	–	0.001	0.000	0.000
EmboTrap	120,265	12,175	3.768	0.536	22,721^§^	0.999	1.000	1.000
Trevo	126,640	–^†^	3.503	–^†^	Dominated by EmboTrap	0.000	0.000	0.000
**Alternative EQ-5D utility scores**								
Solitaire	99,090	–	2.221	–	–	0.000	0.000	0.000
EmboTrap	110,058	10,968	2.615	0.394	27,807^§^	1.000	1.000	1.000
Trevo	116,941	–^†^	2.412	–^†^	Dominated by EmboTrap	0.000	0.000	0.000

To convert from 2022 CNY to 2022 US Dollars ($), divide by 6.96 [37].

† Not reported as Trevo is dominated by EmboTrap.

‡ Due to the number of decimal places presented in the tables for costs and QALYs, the ICERs cannot be manually calculated.

§ ICER is in reference to Solitaire.

¶ Probability represents the proportion of all simulations where the estimated ICER was below the specified cost–effectiveness threshold.

# The base case ICER for Trevo vs Solitaire is 75,751 CNY/QALYs.

CNY: Chinese Yuan; EQ-5D: European Quality of Life 5-dimensional questionnaire; ICER: Incremental cost–effectiveness ratio; QALY: Quality-adjusted life-year.

### Results of sensitivity & scenario analyses

The deterministic OWSA revealed that SR cost had the largest impact on the ICER. Other model drivers identified in the deterministic OWSA included the health utility for mRS 0–2, proportion of patients that achieved functional independence and dependence at 90 days, and the annual discount rate. Regardless of which variable was altered in the deterministic OWSA, EmboTrap was consistently cost-effective compared with both Solitaire and Trevo. In the comparison between Solitaire and Trevo, Trevo was cost-effective when varying all variables other than device cost.

As shown in Table 1, scenario analyses using a younger patient starting age, different time horizons, and alternative health utilities consistently estimated that EmboTrap was cost-effective compared with Solitaire, with ICERs ranging from 22,721 to 66,267 CNY/QALY. Across all scenarios, the ICER for EmboTrap versus Solitaire fell below the three WTP thresholds of interest. Additionally, Trevo was dominated by EmboTrap in all scenarios.

Results of the hypothetical scenario analysis in which the cost of EmboTrap was held constant and the cost of Solitaire was varied demonstrated that when the cost of Solitaire was as low as 0 CNY, EmboTrap was cost-effective at the most conservative WTP threshold of 85,698 CNY/QALY (1 times the GDP per capita), with a resulting ICER of 75,818 CNY/QALY (Supplementary Table 3 & Supplementary Figure 4). When the cost of Trevo was varied, EmboTrap remained either dominant or was cost-effective even when the cost of Trevo was as low as 15,000 CNY (ICER 71,314 CNY/QALY) but was no longer cost-effective at the most conservative WTP threshold of 85,698 CNY/QALY (1 times the GDP per capita) if Trevo cost 10,000 CNY (ICER 93,167 CNY/QALY) (Supplementary Table 3 & Supplementary Figure 4).

**Figure 2.**
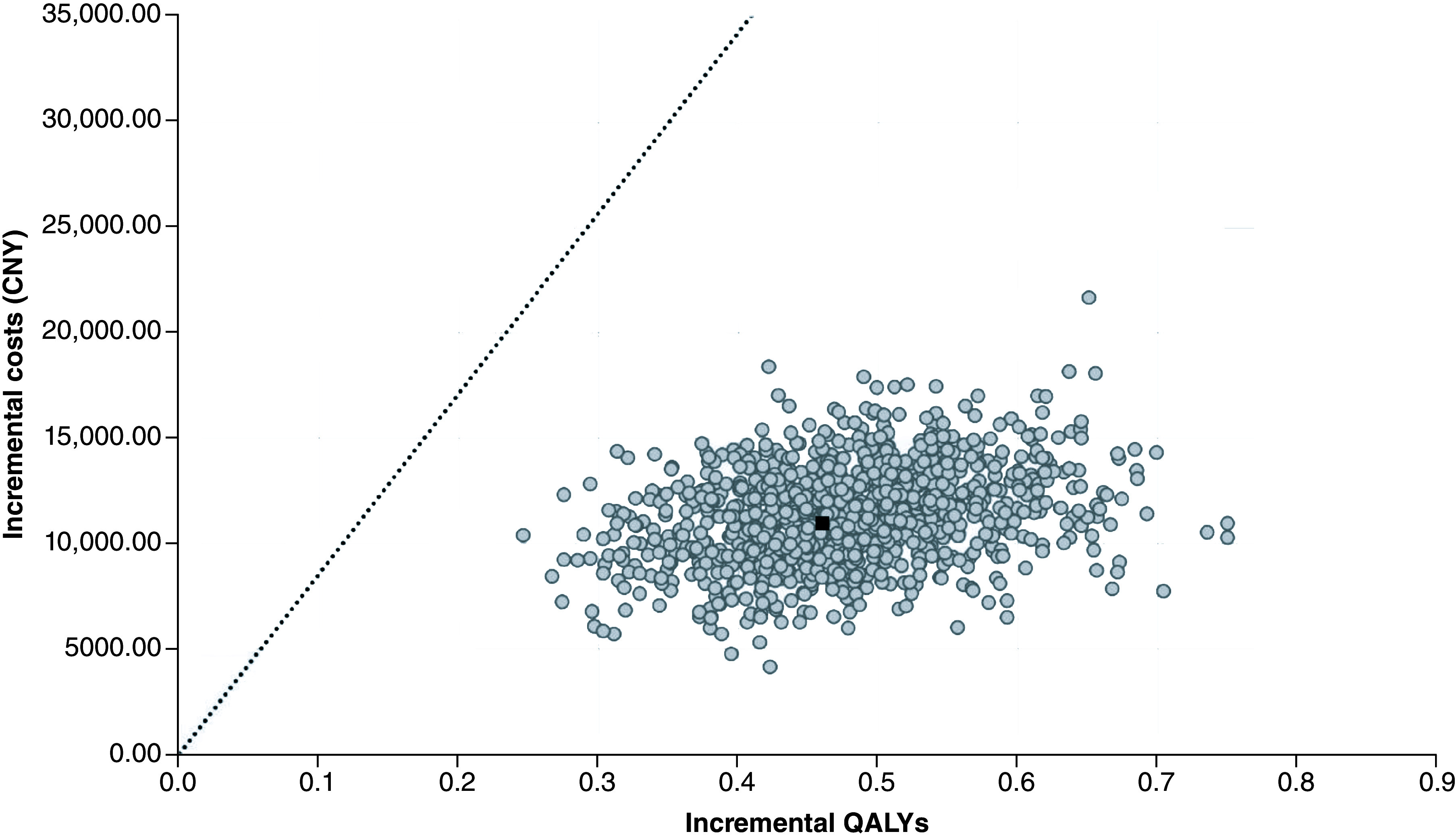
Scatterplot of the probabilistic sensitivity analysis comparing EmboTrap versus Solitaire. To convert from 2022 CNY to 2022 US Dollars ($), divide by 6.96 [37]. Each point represents a simulation. The black square represents the base case (0.464 QALYs gained at an incremental cost of CNY 10,968). The dashed line represents the WTP of 85,698 CNY/QALY. Points on the right side of the dashed line are considered cost-effective. CNY: Chinese Yuan; QALY: Quality-adjusted life-year; WTP: Willingness-to-pay.

The results of the PSAs align with the results of the deterministic analyses (Table 2) with EmboTrap dominating Trevo and being considered cost-effective in relation to Solitaire. When compared with Solitaire, the use of EmboTrap resulted in an ICER of 24,340 CNY/QALY (95% credible interval [CrI]: 14,540 to 35,986) which is less than the 2022 per capita GDP in China of 85,698 CNY/QALY (Figure 2).

**Table 2.** Probabilistic sensitivity analysis results.

Device	Discounted mean costs (2022 CNY) and QALYs per person				
	Mean total cost (CNY) (95% CrI)	Incremental cost (CNY) (95% CrI)	Mean QALYs (95% CrI)	Incremental QALYs (95% CrI)	ICER (CNY/QALY)^‡^ (95% CrI)
Solitaire	96,455 (88,902 to 105,331)	–	2.884 (2.487 to 3.36)	–	–
EmboTrap	107,856 (99,787 to 117,581)	11,401 (6,764 to 15,967)	3.362 (2.907 to 3.927)	0.478 (0.317 to 0.651)	24,340^§^ (14,540 to 35,986)
Trevo	114,694 (106,571 to 124,269)	–^†^	3.126 (2.711 to 3.662)	–^†^	Dominated by EmboTrap

To convert from 2022 CNY to 2022 US Dollars ($), divide by 6.96 [37].

† Not reported as Trevo is dominated by EmboTrap.

‡ As the results are based on 1000 iterations of parametric Monte Carlo simulations, the ICER cannot be manually calculated.

§ ICER is in reference to Solitaire.

CrI: Credible interval; CNY: Chinese Yuan; ICER: Incremental cost–effectiveness ratios; QALY: Quality-adjusted life.

**Figure 3.**
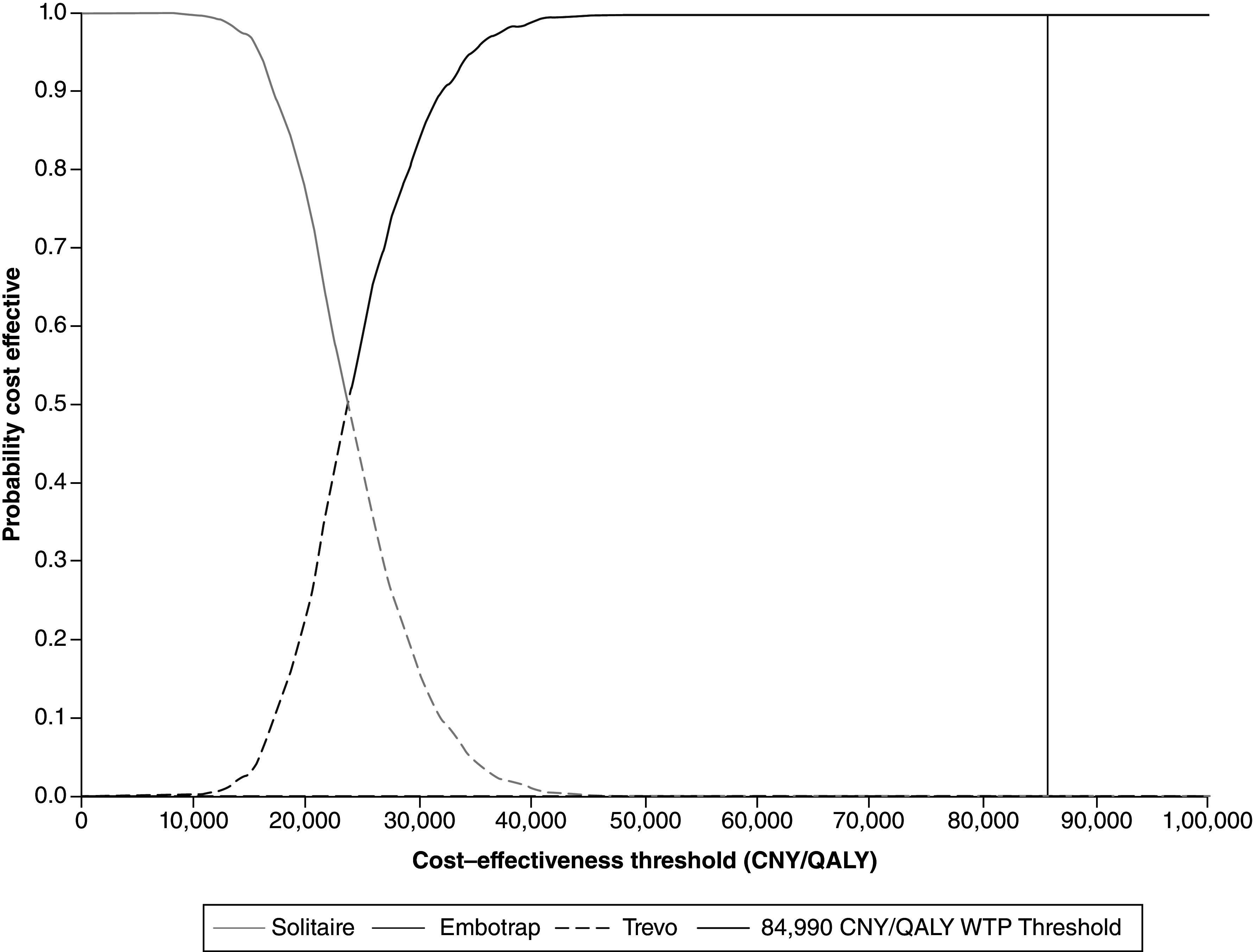
Cost–effectiveness acceptability curve. To convert from 2022 CNY to 2022 US Dollars ($), divide by 6.96 [37]. Trevo is never the most cost-effective device. CNY: Chinese Yuan; QALY: Quality-adjusted life-year; WTP: Willingness-to-pay.

The probability of EmboTrap, Solitaire, or Trevo being cost-effective at different WTP thresholds is outlined in Table 1. The cost–effectiveness acceptability curve (Figure 3) demonstrated that at a WTP threshold of 23,615 CNY/QALY, EmboTrap had a 50% chance of being cost-effective (versus Solitaire). For WTP thresholds exceeding 23,615 CNY/QALY, EmboTrap had a higher probability of being cost-effective compared with Solitaire. Trevo had a 0% probability of being the cost-effective device across all WTP thresholds (Figure 3).

## Discussion

The aim of the current study was to assess the cost–effectiveness of EmboTrap versus Trevo versus Solitaire in China based on the proportion of patients with AIS who achieved good functional outcomes as reported in MASTRO I.

The results of this study suggest that EmboTrap is the dominant treatment option relative to Trevo and is cost-effective compared with Solitaire in relation to the three WTP thresholds considered. These results leverage the key finding from MASTRO I that the choice of SR in the treatment of AIS may impact functional outcomes and quantify potential long-term healthcare cost impact, both of which are important considerations given the substantial economic burden of stroke in China. In alignment with this, the Government of China has incorporated stroke prevention and treatment into the “Healthy China 2030” program [34], aiming to increase the stroke prevention and treatment network. Under this policy environment, it will be critical to utilize medical devices that offer value for money to benefit more patients in need.

In addition to reducing long-term costs, achieving better functional outcomes post-MT resulted in higher QALYs and is therefore impactful from a population health perspective. Based on the proportion of patients who achieved mRS 0–2 and mRS 3–5 in MASTRO I [9], the current analysis demonstrated that MT using EmboTrap was associated with an increase in QALYs over the 10-year time horizon compared with Solitaire and Trevo. Considering that health-related QoL is diminished in survivors of stroke compared with individuals who have not experienced a stroke [35], increasing QALYs post-stroke positively impacts patient well-being by improving the overall quality of the years lived post-stroke and subsequently increasing health-related QoL (HRQoL). Furthermore, increased HRQoL post-stroke can improve physical and psychological functioning [35,36].

The robustness of the base case results was supported by the results of the deterministic OWSA, PSAs and multiple scenario analyses, with EmboTrap consistently dominating Trevo and remaining cost-effective compared with Solitaire. The deterministic OWSA revealed that device cost had the largest impact on the results. The results of the PSA confirm the robustness of the deterministic results, demonstrating that EmboTrap dominates Trevo and is cost-effective relative to Solitaire in 100% of probabilistic iterations at all assessed WTP thresholds. However, these results should be interpreted with caution, as the PSA only accounts for uncertainty related to the parameters included in the model. Structural (model) uncertainty in addition to uncertainty pertaining to the sources of costs and utilities, remain unaddressed. Implementing a 5- or 15-year time horizon and using a starting patient age of 65 years each had a minimal impact on the model results. Similarly, using alternative utility values that were lower than those used in the base case also had a minimal impact on the model results; however, the results of this scenario analysis should be interpreted with caution since the utility values were sourced from a 2014 study examining HRQoL in patients with transient ischemic attack or minor ischemic stroke and may not be applicable to current clinical practice for the population interest [29,32]. When comparing Solitaire and EmboTrap, the largest change in ICER relative to the base case occurred when exploring a one-year time horizon. This result was expected since the majority of patients are still alive but have not accrued the long-term benefit associated with good functional outcomes post-MT with EmboTrap; namely, improved HRQoL and lower total costs (compared with patients who did not achieve good functional outcomes). In other words, the MT procedure is a one-off, front-loaded cost with a long-term benefit. While the results of a 5- or 15-year time horizon support the robustness of the analyses, a 10-year time horizon adequately captures the long-term HRQoL and cost benefits afforded by using EmboTrap and appropriately represents the current life expectancy in China of approximately 78 years [15].

Economic evaluations play a critical role in health technology reimbursement [31] and the use of WTP thresholds is instrumental in guiding reimbursement decisions to optimize value for money. Although the Chinese government has not explicitly specified a WTP threshold, the 2020 China Guidelines for Pharmacoeconomic Evaluations advised that ICERs below 1 to 3-times the national GDP per capita are considered cost-effective [17]. The results of this study suggest that Trevo is dominated by EmboTrap and that EmboTrap is cost-effective in relation to Solitaire (ICER of 23,615 CNY/QALY) when considering the range of WTP thresholds based on the GDP per capita (85,698 CNY/QALY to 257,084 CNY/QALY).

As SR cost was the largest model driver, changes to device cost are expected to influence the overall cost–effectiveness of the devices. In consideration of potential cost reductions, the results of the deterministic OWSA indicated that reductions in the cost of EmboTrap or the annual cost of post-stroke care (for mRS 0–2 or mRS 3–5) further reduced the ICER in relation to Solitaire. In comparison to Trevo, results of the deterministic OWSA also indicated that reductions in the cost of EmboTrap or the annual cost of post-stroke care (for mRS 0–2) further increased the cost–effectiveness of EmboTrap by increasing the difference in device and post-stroke care costs. Device cost was also varied in the PSA, which did not impact the overall trends or conclusions in the base case analysis. Finally, the hypothetical scenario analysis exploring the cost–effectiveness of EmboTrap when the device cost of Solitaire and Trevo were varied demonstrated that EmboTrap is cost-effective even when Solitaire is not associated with any cost and when the cost of Trevo was as low as 15,000 CNY. In this hypothetical scenario analysis, cost–effectiveness was assessed relative to the most conservative WTP threshold of 85,698 CNY/QALY (1-times the GDP per capita). Although the deterministic OWSA found the model to be most sensitive to device cost, in the context of the current analysis, the ICERs for EmboTrap are robust and stable relative to potential changes in competitor pricing. Taken together, these results suggest that despite the model's sensitivity to device cost, the results are robust to such fluctuations.

The economic benefits of EmboTrap demonstrated in the current analysis are due to a higher proportion of patients achieving good functional outcomes (i.e., mRS 0–2) relative to Solitaire or Trevo, as demonstrated in MASTRO I [9]. There were several indicators of reasons for this difference in MASTRO I, including patient selection, technique (BGC usage) and device design. This was substantiated by the enhanced recanalization ability of EmboTrap relative to Solitaire and Trevo, as demonstrated by numerically higher rates of complete recanalization (first-pass recanalization (FPR) modified treatment in cerebral infarction [mTICI] ≥2c) and successful recanalization (FPR mTICI ≥2b) on the first pass, and final complete (thrombolysis in cerebral infarction [TICI] 3) and final successful (mTICI ≥2b) recanalization [9]. This is in-line with a systematic pre-clinical *in vitro* comparison showing EmboTrap's superior ability to remove clots [10]. Furthermore, EmboTrap was associated with higher rates of successful reperfusion when compared with a performance goal derived from previous Solitaire and Trevo trials [11]. This emphasizes the role of device design in achieving improved functional outcomes. The functionality of a SR is strongly influenced by its geometric design [8] and, unlike Solitaire and Trevo, which are second-generation nitinol stents with a closed cell peak-peak geometric configuration, EmboTrap is a third-generation two-layer nitinol stent [8]. EmboTrap is composed of an inner closed cell layer that is surrounded by an outer layer with open-cell configuration on the proximal end and closed-cell configuration on the distal end [8]. The difference in device design between EmboTrap, Solitaire and Trevo may account for the observed differences in recanalization rates, which subsequently impacts the functional and economic outcomes associated with the SRs. Taken together, the use of EmboTrap during MT may improve clinical outcomes due to improved device functionality, subsequently reducing the economic burden of stroke.

### Strengths & limitations

There are several strengths associated with this study. A thorough review was undertaken to ensure the model, inputs, and outcome were applicable to the current landscape, and to understand the local implications of the findings. This review enhanced the robustness and applicability of the results by allowing for a thorough understanding of both the strengths and weaknesses of the analyses. Moreover, adherence to the 2020 China Guidelines for Pharmacoeconomic Evaluations [17] and the CHEERS statement [16] ensured the analyses were conducted and the findings were reported in an appropriate manner. Furthermore, the current study used a model structure that has been validated in a previous health technology assessment [21], as such, the methodological approach is robust, and the findings can be considered reliable. Lastly, model inputs were based on recent clinical and cost data sourced from published literature or cost databases, thereby accurately reflecting the market in China for the treatment of AIS.

As with all modelling studies, the current study has several limitations that should be taken into consideration when interpreting the results. Despite model inputs being sourced from best available data, assumptions were required relating to model cost inputs. Cost inputs used in the model may not fully capture the most recent and innovative treatment patterns in China as initial hospitalization costs were sourced from a study that assessed the cost of stroke from 2017 to 2018 [27] and the annual cost of post-stroke care was sourced from a 2018 study that reported costs based on the China National Stroke Registry [29]. However, the cost inputs reflect the best-available data representing the market in China for treatment of AIS. Similarly, assumptions were required relating to patient distributions in the decision tree and the transition probabilities between Markov health states. The proportion of patients in each state (mRS 0–2, mRS 3–5 and mRS 6) in the decision tree may not be generalizable to patients in China as these proportions were sourced from MASTRO I which had a global focus with respect to the SLR inclusion criteria [9]. Furthermore, as the 2019 update on management of ischemic cerebrovascular diseases released by the Chinese Stroke Association suggests that the operator may choose to use other thrombectomy or aspiration devices during MT [6], these proportions may not be generalizable across operators and/or hospitals in China. Long-term transition probabilities were adapted from a health technology assessment that used five-year survival data from the Oxford Vascular Study which was a UK-based study, and therefore could limit generalizability to Chinese patients [21]. Additionally, as there are regional health authorities in China, post-stroke healthcare may vary across regions which could impact the disease course and long-term recovery thereby influencing transitions between model health states. Despite the limitations in terms of generalizability, the proportion of patients in the decision tree and transition probabilities were sourced using best-available data as a paucity of published values was noted. Given the dearth of published values, further research exploring the up-to-date costs associated with stroke is needed to inform future cost–effectiveness models.

Another potential limitation was that the cost–effectiveness analysis assumed that only SRs would be used during MT and did not account for the use of adjunctive devices (e.g., IV-tPA and balloon guide catheters) or differences in MT techniques. Similarly, the procedural costs assume that no rescue devices were used, and the model does not account for costs and changes in health status that are associated with recurrent stroke. However, it is important to note these limitations are due to the population of the current study being based on MASTRO I, in which many of the included studies did not report this level of detail. Furthermore, MASTRO I included studies from 2015 to 2022 which may not reflect outcomes achieved using the relevant SRs applicable to current clinical practice [9]. Finally, while a WTP threshold ranging from 1 to 3-times China's GDP per capita was assumed based on a recent publication [31], this threshold may not be accurate and could impact the assumptions around the cost–effectiveness of EmboTrap. While accounting for adjunctive devices, rescue devices, changes in health after recurrent stroke, clot composition, operator experience and hospital volume may influence the economic outcomes associated with SR use, assessing this was beyond the scope of this study. Further research is needed to understand how including these factors may impact mRS, and subsequently cost–effectiveness, following MT. Despite these limitations, the best-available data were used to inform the patient cohort inputs, SR use in current clinical practice, and the appropriate WTP threshold for assessing cost–effectiveness.

Overall, this study sought to assess the cost–effectiveness of EmboTrap versus Trevo versus Solitaire based on 90-day post-stroke functional outcomes and their subsequent economic impact following MT. The results of MASTRO I suggest that SR choice may impact post-stroke outcomes, with EmboTrap being associated with increased likelihood of achieving functional independence compared with Solitaire and Trevo [9]. The current study suggests that EmboTrap dominates Trevo and is cost-effective in relation to Solitaire, when considering WTP thresholds of 1, 1.5 and 3-times the China GDP per capita. The results of the current analysis emphasize the importance of SR selection for MT. In addition to considering factors such as vascular and thrombotic conditions, surgeon preference, and institutional factors, when possible, selecting a SR that optimizes outcomes post-stroke should be an important consideration for both clinicians and healthcare payers alike.

## Conclusion

The results of this study suggest that MT with EmboTrap dominates Trevo and is cost-effective in relation to Solitaire in the treatment of patients with AIS in China. Considering that patient 90-day mRS score is predictive of long-term costs and HRQoL, in addition to accounting for typical SR selection factors, selecting a SR associated with good functional outcomes 90-days post-stroke is an important consideration from a clinical and healthcare payer perspective.

## Summary points

- A cost–effectiveness analysis was conducted for three commonly used SRs (EmboTrap, Solitaire and Trevo) using functional outcome data reported in MASTRO I, a recent living systematic literature review and meta-analysis.
- Over a 10-year time horizon, Trevo was dominated by EmboTrap as Trevo was more costly but less effective.
- EmboTrap was cost-effective relative to Solitaire based on WTP thresholds of 1, 1.5 and 3-times the China GDP per capita (i.e., 85,698 CNY/QALY, 128,547 CNY/QALY and 257,084 CNY/QALY, respectively).
- Multiple scenario analyses and the PSA support the results of the base case analyses, with EmboTrap dominating Trevo and being cost-effective compared with both Solitaire at all three WTP thresholds of interest (85,698 CNY/QALY, 128,547 CNY/QALY and 257,084 CNY/QALY).
- The deterministic OWSA served to identify key model drivers and supported the robustness of the results; importantly, varying device cost by ±10% maintained similar trends with the base case analysis, with EmboTrap dominating Trevo and being cost-effective compared with Solitaire.
- The results of this study should be interpreted within the context of the associated limitations and assumptions related to model inputs and calculations.
- Further research exploring the costs associated with stroke is needed to inform future cost–effectiveness models.
- The results of this analysis provides additional information that payers and clinicians may leverage in making evidence-based decisions to inform SR selection for use in MT.
- The results from the current study suggest that outcomes 90-days post-stroke are predictive of long-term costs and HRQoL, as such, selecting a SR associated with good functional outcomes 90-days post-stroke is an important consideration from a clinical and healthcare payer perspective.

## Supplementary Material



## References

[1] Tsao CW, Aday AW, Almarzooq ZI Heart disease and stroke statistics-2022 update: a report from the american heart association. Circulation 145(8), e153–e639 (2022). 35078371 10.1161/CIR.0000000000001052

[2] Tu WJ, Wang LD. China stroke surveillance report 2021. Mil. Med. Res. 10(1), 33 (2023). 37468952 10.1186/s40779-023-00463-x PMC10355019

[3] Tu WJ, Zhao Z, Yin P Estimated Burden of stroke in China in 2020. JAMA Netw. Open. 6(3), e231455 (2023). 36862407 10.1001/jamanetworkopen.2023.1455 PMC9982699

[4] Wang YJ, Li ZX, Gu HQ China stroke statistics 2019: a report from the National Center for Healthcare Quality Management in Neurological Diseases, China National Clinical Research Center for Neurological Diseases, the Chinese Stroke Association, National Center for Chronic and Non-communicable Disease Control and Prevention, Chinese Center for Disease Control and Prevention and Institute for Global Neuroscience and Stroke Collaborations. Stroke Vasc. Neurol. 5(3), 211–239 (2020). 32826385 10.1136/svn-2020-000457 PMC7548521

[5] Miao Z, Huo X, Gao F Endovascular therapy for Acute ischemic Stroke Trial (EAST): study protocol for a prospective, multicentre control trial in China. Stroke Vasc. Neurol. 1(2), 44–51 (2016). 28959463 10.1136/svn-2016-000022 PMC5435194

[6] Liu L, Chen W, Zhou H Chinese Stroke Association guidelines for clinical management of cerebrovascular disorders: executive summary and 2019 update of clinical management of ischaemic cerebrovascular diseases. Stroke Vasc. Neurol. 5(2), 159–176 (2020). 32561535 10.1136/svn-2020-000378 PMC7337371

[7] Tsang COA, Cheung IHW, Lau KK Outcomes of stent retriever versus aspiration-first thrombectomy in ischemic stroke: a systematic review and meta-analysis. AJNR Am. J. Neuroradiol. 39(11), 2070–2076 (2018). 30337435 10.3174/ajnr.A5825 PMC7655372

[8] Ahmed SU, Chen X, Peeling L, Kelly ME. Stentrievers: an engineering review. Interv. Neuroradiol. 29(2), 125–133 (2022). 35253526 10.1177/15910199221081243 PMC10152824

[9] Zaidat OO, Ikeme S, Sheth SA MASTRO I: Meta-Analysis and Systematic Review of thrombectomy stent retriever outcomes: comparing functional, safety and recanalization outcomes between EmboTrap, Solitaire and Trevo in acute ischemic stroke. J. Comp. Eff. Res. 12(5), e230001 (2023). 37039285 10.57264/cer-2023-0001 PMC10402757

[10] Mirza M, McCarthy R, Gilvarry M. EP64 systematic review and analysis of pre-clinical side-by-side comparisons of EmboTrap versus Solitaire performance. J. Neurointerv. Surg. 13(Suppl. 2), A26 (2021).

[11] Zaidat OO, Bozorgchami H, Ribó M Primary results of the multicenter ARISE II study (analysis of revascularization in ischemic stroke with EmboTrap). Stroke 49(5), 1107–1115 (2018). 29643261 10.1161/STROKEAHA.117.020125

[12] Askew RL, Capo-Lugo CE, Sangha R, Naidech A, Prabhakaran S. Trade-offs in quality-of-life assessment between the modified rankin scale and neuro-QoL measures. Value Health 23(10), 1366–1372 (2020). 33032781 10.1016/j.jval.2020.06.011 PMC7547147

[13] Kim SE, Lee H, Kim JY Three-month modified Rankin Scale as a determinant of 5-year cumulative costs after ischemic stroke: an analysis of 11,136 patients in Korea. Neurology 94(9), e978–e991 (2020). 32029544 10.1212/WNL.0000000000009034

[14] Dewilde S, Annemans L, Peeters A Modified Rankin scale as a determinant of direct medical costs after stroke. Int. J. Stroke 12(4), 392–400 (2017). 28164742 10.1177/1747493017691984

[15] Wang L, Yu Y, Zhou L Endovascular treatment for basilar artery occlusion: a cost–effectiveness analysis based on a meta-analysis. Front Neurol. 14, 1267554 (2023). 37928158 10.3389/fneur.2023.1267554 PMC10623329

[16] Husereau D, Drummond M, Petrou S Consolidated Health Economic Evaluation Reporting Standards (CHEERS) statement. Value Health 16(2), e1–e5 (2013). 23538200 10.1016/j.jval.2013.02.010

[17] China Guidelines for Pharmacoeconomic Evaluations (2020). Available at: https://www.ispor.org/heor-resources/more-heor-resources/pharmacoeconomic-guidelines/pe-guideline-detail/china-mainland (Accessed: January 2023).

[18] R Core Team. R: A Language and Environment for Statistical Computing. R Foundation for Statistical Computing, Vienna, Austria, Available at: https://www.r-project.org/ (Accessed: 2024).

[19] Ye Q, Zhai F, Chao B Rates of intravenous thrombolysis and endovascular therapy for acute ischaemic stroke in China between 2019 and 2020. Lancet Reg. Health West Pac. 21, 100406 (2022). 35243459 10.1016/j.lanwpc.2022.100406 PMC8873940

[20] Press CS. 7th National Population Census. Available at: http://www.stats.gov.cn/sj/pcsj/rkpc/7rp/zk/indexch.htm (Accessed: May 2023).

[21] Health Quality Ontario. Mechanical thrombectomy in patients with acute ischemic stroke: a health technology assessment. Ont. Health Technol. Assess Ser. 16(4), 1–79 (2016). PMC4761918 27026799

[22] Luengo-Fernandez R, Paul NL, Gray AM Population-based study of disability and institutionalization after transient ischemic attack and stroke: 10-year results of the Oxford Vascular Study. Stroke 44(10), 2854–2861 (2013). 23920019 10.1161/STROKEAHA.113.001584 PMC4946627

[23] Luengo-Fernandez R, Gray AM, Bull L Quality of life after TIA and stroke: ten-year results of the Oxford Vascular Study. Neurology 81(18), 1588–1595 (2013). 24107865 10.1212/WNL.0b013e3182a9f45f PMC3806919

[24] Vanni T, Karnon J, Madan J Calibrating models in economic evaluation: a seven-step approach. Pharmacoeconomics 29(1), 35–49 (2011). 21142277 10.2165/11584600-000000000-00000

[25] Press CS. National Bureau of Statistics of China. China Statistical Yearbook. Available at: https://data.stats.gov.cn/publish.htm?sort=1 (Accessed: May 2023).

[26] Taimao Medical Device Tendering Network. Available at: https://www.ylqxzb.com/ (Accessed: August 2023).

[27] Lv W, Wang A, Wang Q One-year direct and indirect costs of ischaemic stroke in China. Stroke Vasc. Neurol. 9(4), 380–389 (2024). 37788911 10.1136/svn-2023-002296 PMC11420908

[28] Meng X, Wang A, Wang R EE208 Direct Cost and Indirect Cost of Ischemic Stroke in China. Value Health 25(12), S93–S94 (2022).

[29] Pan Y, Cai X, Huo X Cost–effectiveness of mechanical thrombectomy within 6 hours of acute ischaemic stroke in China. BMJ Open 8(2), e018951 (2018). 10.1136/bmjopen-2017-018951 PMC5855394 29472264

[30] Wang X, Moullaali TJ, Li Q Utility-Weighted Modified Rankin Scale Scores for the Assessment of Stroke Outcome: Pooled Analysis of 20 000+ Patients. Stroke 51(8), 2411–2417 (2020). 32640944 10.1161/STROKEAHA.119.028523

[31] Cai D, Shi S, Jiang S Estimation of the cost-effective threshold of a quality-adjusted life year in China based on the value of statistical life. Eur. J. Health Econ. 23(4), 607–615 (2022). 34655364 10.1007/s10198-021-01384-z PMC9135816

[32] Wang YL, Pan YS, Zhao XQ Recurrent stroke was associated with poor quality of life in patients with transient ischemic attack or minor stroke: finding from the CHANCE trial. CNS Neurosci. Ther. 20(12), 1029–1035 (2014). 25307297 10.1111/cns.12329 PMC6493002

[33] Drummond MF, Sculpher MJ, Claxton K, Stoddart GL, Torrance GW. Methods for the Economic Evaluation of Health Care Programmes – (Fourth Edition). Oxford University Press, Oxford, UK (2015).

[34] Chao BH, Tu WJ, Wang LD, Ni J. Initial establishment of a stroke management model in China: 10 years (2011–2020) of Stroke Prevention Project Committee, National Health Commission. Chin. Med. J. 134(20), 2418–2420 (2021). 34620751 10.1097/CM9.0000000000001856 PMC8654431

[35] Chen Q, Cao C, Gong L, Zhang Y. Health related quality of life in stroke patients and risk factors associated with patients for return to work. Medicine (Baltimore) 98(16), e15130 (2019). 31008934 10.1097/MD.0000000000015130 PMC6494282

[36] Ramos-Lima MJM, Brasileiro IC, Lima TL, Braga-Neto P. Quality of life after stroke: impact of clinical and sociodemographic factors. Clinics (Sao Paulo) 73, e418 (2018). 30304300 10.6061/clinics/2017/e418 PMC6152181

[37] Bank of China. December 31st, 2022 Exchange Rates. Available at: https://www.boc.cn/sourcedb/whpj/enindex2.htm

