# Supplementary material for: The economic impact associated with stent retriever selection for the treatment of acute ischemic stroke: a cost–effectiveness analysis of MASTRO I data from a Chinese healthcare system perspective
Source: J Comp Eff Res. 2024 Nov 5;13(11):e240160. doi: 10.57264/cer-2024-0160 (PMC11542088; doi:10.57264/cer-2024-0160)
Supplement: Supplementary file 1 [file cer-13-240160-s1.docx]

# Supplementary tables and Figures

Supplementary Table 1. Transition probabilities

| **Input Variables** | **Base case** | **Range** | **Distribution** | **Reference** |
| --- | --- | --- | --- | --- |
| **Proportion of Patients in each state 90 days** |  |  | Multinomial | [[9](#_ENREF_9)] |
| *EmboTrap* |  |  |  |  |
| Functional independence (mRS=0-2) | 0.574 | 0.517-0.631 |  |  |
| Functional dependence (mRS=3-5) | 0.314 | 0.272- 0.356 |  |  |
| Death (mRS=6) | 0.112 | 0.097-0.127 |  |  |
| *Trevo* |  |  |  |  |
| Functional independence (mRS=0-2) | 0.500 | 0.450-0.550 |  |  |
| Functional dependence (mRS=3-5) | 0.355 | 0.320-0.391 |  |  |
| Death (mRS=6) | 0.145 | 0.131- 0.160 |  |  |
| *Solitaire* |  |  |  |  |
| Functional independence (mRS=0-2) | 0.453 | 0.408-0.498 |  |  |
| Functional dependence (mRS=3-5) | 0.343 | 0.315- 0.371 |  |  |
| Death (mRS=6) | 0.204 | 0.187-0.221 |  |  |
| **Transition probabilities after 90 days** |  |  |  | [[21](#_ENREF_21)] |
| Functional independence to functional dependence |  |  |  |  |
| 4-6 months | 0.032 | 0.028-0.036 | Beta |  |
| 7-12 months | 0.022 | 0.019-0.023 | Beta |  |
| 13-24 months | 0.013 | 0.013-0.017 | Beta |  |
| 25-36 months* | 0.011 | - | - |  |
| 37-48 months* | 0.009 | - | - |  |
| 49-60 months* | 0.008 | - | - |  |
| 60-120 months | 0 | - | - |  |
| Functional dependence to functional independence |  |  |  |  |
| 4-6 months | 0.037 | 0.034-0.041 | Log normal |  |
| 7-12 months | 0.015 | 0.014-0.017 | Log normal |  |
| 13+ months** | 0 | - | - |  |
| **Mortality after 90 days***** |  |  | Log normal | [[20](#_ENREF_20), [21](#_ENREF_21)] |
| RR mortality 4-12 months, functional independence | 2.646 | 2.1-2.9 |  |  |
| RR mortality 13+ months, functional independence | 1.035 | 1.0-1.1 |  |  |
| RR mortality 4-12 months, functional dependence | 7.570 | 7.5-8.2 |  |  |
| RR mortality 13+ months, functional dependence | 2.899 | 2.6-3.0 |  |  |

*Not varied in probabilistic sensitivity analysis.

**After one-year post-stroke patients could not transition to functional independence.

***Monthly risk of mortality calculated by multiplying the RR by the age-specific all-cause mortality for the Chinese population.

Abbreviations: mRS = modified Rankin Scale; RR = risk ratio.

Supplementary Table 2. Cost and Utility Model Inputs

| **Input Parameters** | **Base case** | **Range** | **Distribution** | **Reference** |
| --- | --- | --- | --- | --- |
| **Cost Input Parameters** | | | | |
| **SR Cost (Percent Increase Relative to EmboTrap)** |  |  | Gamma | [[26](#_ENREF_26)] |
| EmboTrap | - | - |  |  |
| Trevo | 28.2% | 22.0% - 41.7% |  |  |
| Solitaire | -18.6% | -30.6% - 19.0% |  |  |
| **Cost for initial hospitalization (2022 CNY)** |  |  | Gamma | [[27-29](#_ENREF_27)] |
| Functional independence (mRS 0-2) | 17,854 | 16,068 - 19,639 |  |  |
| Functional dependence (mRS 3-5) | 27,210 | 24,489 - 29,930 |  |  |
| Death (mRS 6) | 19,746 | 17,772 - 21,721 |  |  |
| **Annual cost of post-stroke care (2022 CNY)** |  |  | Gamma | [[29](#_ENREF_29)] |
| Functional independence (mRS 0-2) | 8,386 | 7,548 - 9,225 |  |  |
| Functional dependence (mRS 3-5) | 12,245 | 11,020 - 13,469 |  |  |
| **Utility Input Parameters** | | | | |
| **Utility weights** |  |  | Beta | [[30](#_ENREF_30)] |
| Functional independence (mRS 0-2) | 0.867 | 0.780 - 0.954 |  |  |
| Functional dependence (mRS 3-5) | 0.335 | 0.302- 0.369 |  |  |
| **Utility weights (Scenario analysis)** |  |  | Beta | [[29](#_ENREF_29), [32](#_ENREF_32)] |
| Functional independence (mRS 0-2) | 0.760 | 0.690 - 0.820 |  |  |
| Functional dependence (mRS 3-5) | 0.210 | 0.170 - 0.260 |  |  |

Note: To convert from 2022 CNY to 2022 United States Dollars ($), divide by 6.96 [37].

Abbreviations: CNY = Chinese Yuan; mRS = modified Rankin Scale; SR = stent retriever.

Supplementary Table 3. ICERs Associated with Varying Cost of Solitaire and Trevo

| **Cost of Comparator Device (CNY)** | **ICER of EmboTrap Versus Comparator Device**  **(CNY/QALY)** |
| --- | --- |
| **Solitaire** | |
| 0 | 75,818 |
| 1,000 | 73,665 |
| 2,500 | 70,435 |
| 5,000 | 65,052 |
| 7,500 | 59,670 |
| 10,000 | 54,287 |
| 12,500 | 48,905 |
| 15,000 | 43,522 |
| 17,500 | 38,140 |
| 20,000 | 32,757 |
| 22,500 | 27,374 |
| **Trevo** | |
| 0 | 136,873 |
| 2,500 | 125,947 |
| 5,000 | 115,020 |
| 10,000 | 93,167 |
| 15,000 | 71,314 |
| 20,000 | 49,462 |
| 25,000 | 27,609 |
| 27,500 | 16,682 |
| 30,000 | 5,756 |
| 32,500 | EmboTrap is dominant |
| 35,000 | EmboTrap is dominant |
| 37,500 | EmboTrap is dominant |

Note: To convert from 2022 CNY to 2022 United States Dollars ($), divide by 6.96 [37].

Abbreviations: CNY = Chinese Yuan; ICER = incremental cost-effectiveness ratio; QALY = quality-adjusted life-year.

Supplementary Figure 1. Tornado Diagram for the Pairwise Deterministic One-Way Sensitivity Analysis of EmboTrap Versus Solitaire


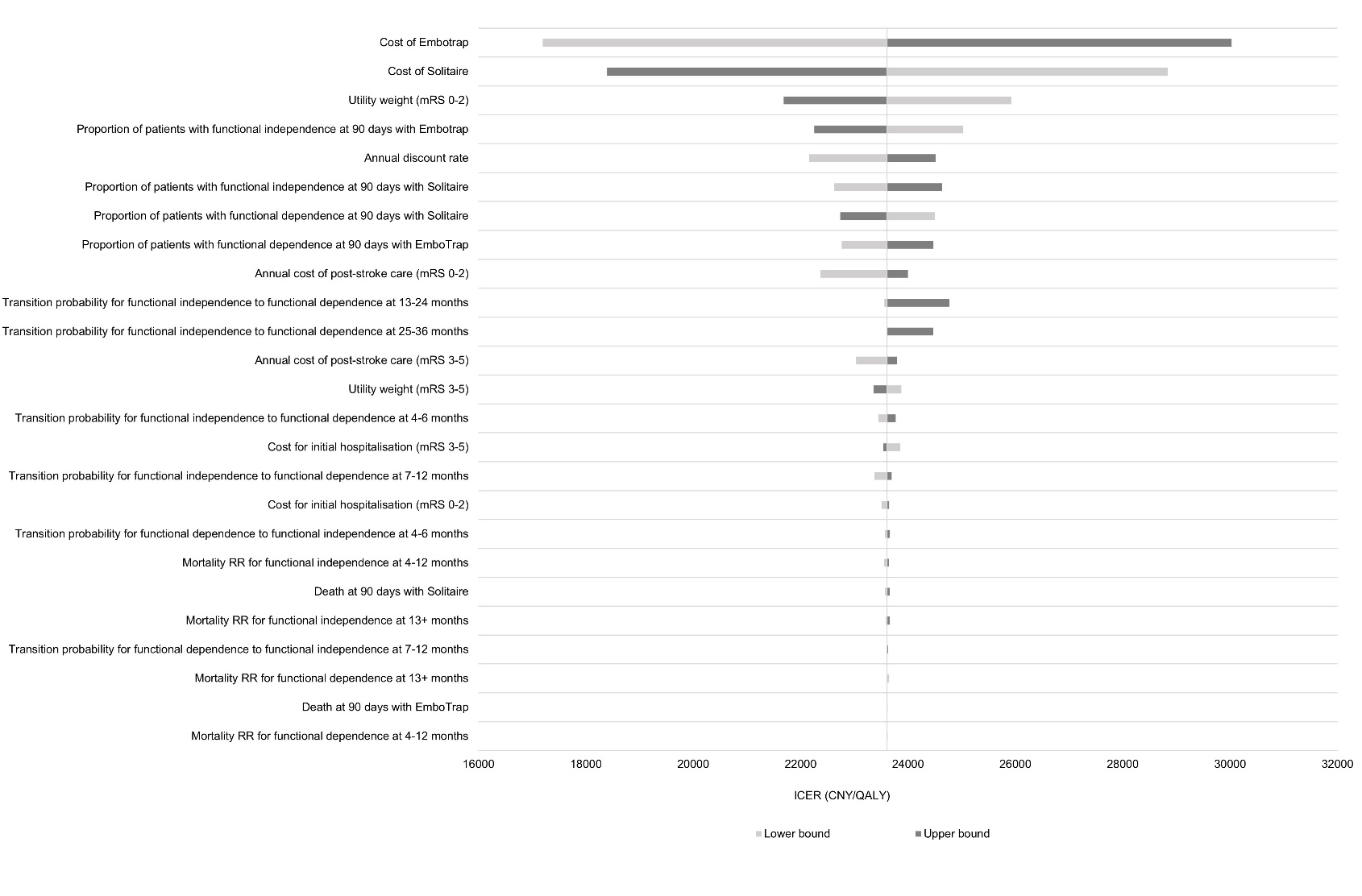


Note: To convert from 2022 CNY to 2022 United States Dollars ($), divide by 6.96 [37].

Abbreviations: CNY = Chinese Yuan; ICER = incremental cost-effectiveness ratio; mRS = modified Rankin Scale; QALY = quality-adjusted life-year.

Supplementary Figure 2. Tornado Diagram for the Pairwise Deterministic One-Way Sensitivity Analysis of Trevo Versus EmboTrap


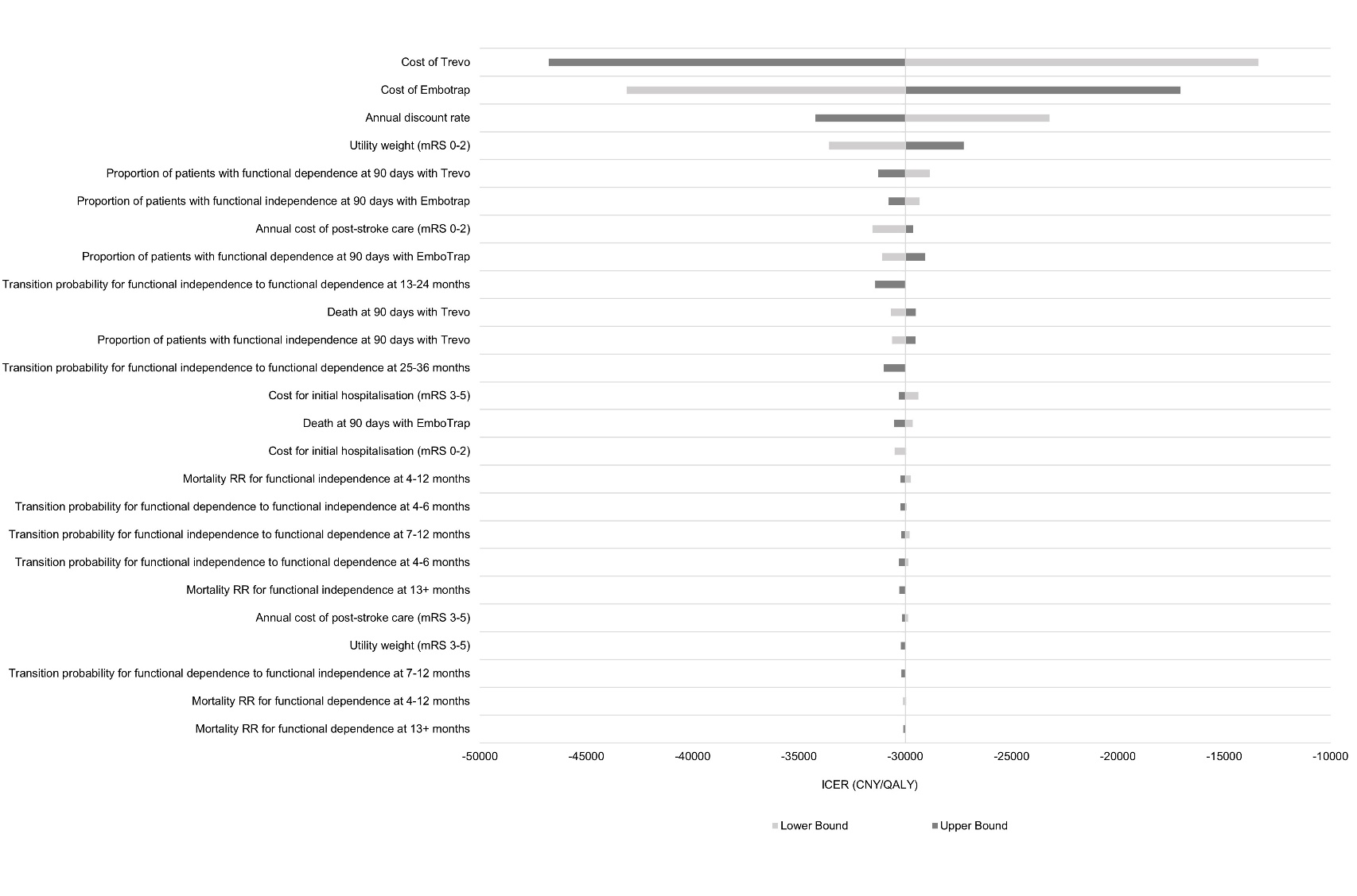


Note: To convert from 2022 CNY to 2022 United States Dollars ($), divide by 6.96 [37].

Abbreviations: CNY = Chinese Yuan; ICER = incremental cost-effectiveness ratio; mRS = modified Rankin Scale; QALY = quality-adjusted life-year.

Supplementary Figure 3. Tornado Diagram for the Pairwise Deterministic One-Way Sensitivity Analysis of Trevo Versus Solitaire

**
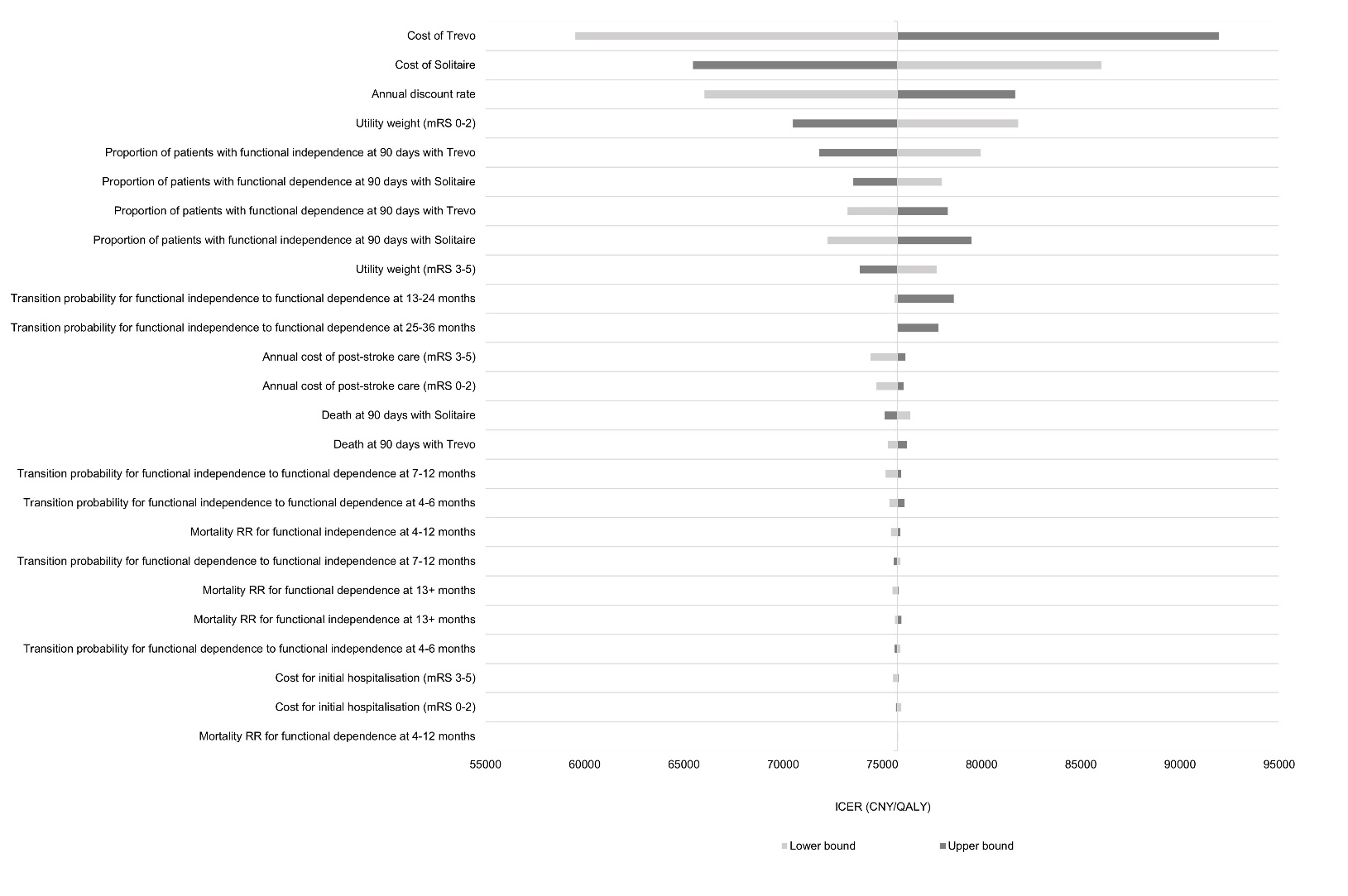
**

Note: To convert from 2022 CNY to 2022 United States Dollars ($), divide by 6.96 [37].

Abbreviations: CNY = Chinese Yuan; ICER = incremental cost-effectiveness ratio; mRS = modified Rankin Scale; QALY = quality-adjusted life-year.

Supplementary Figure 4. ICERs Associated with Varying Cost of (A) Solitaire and (B) Trevo


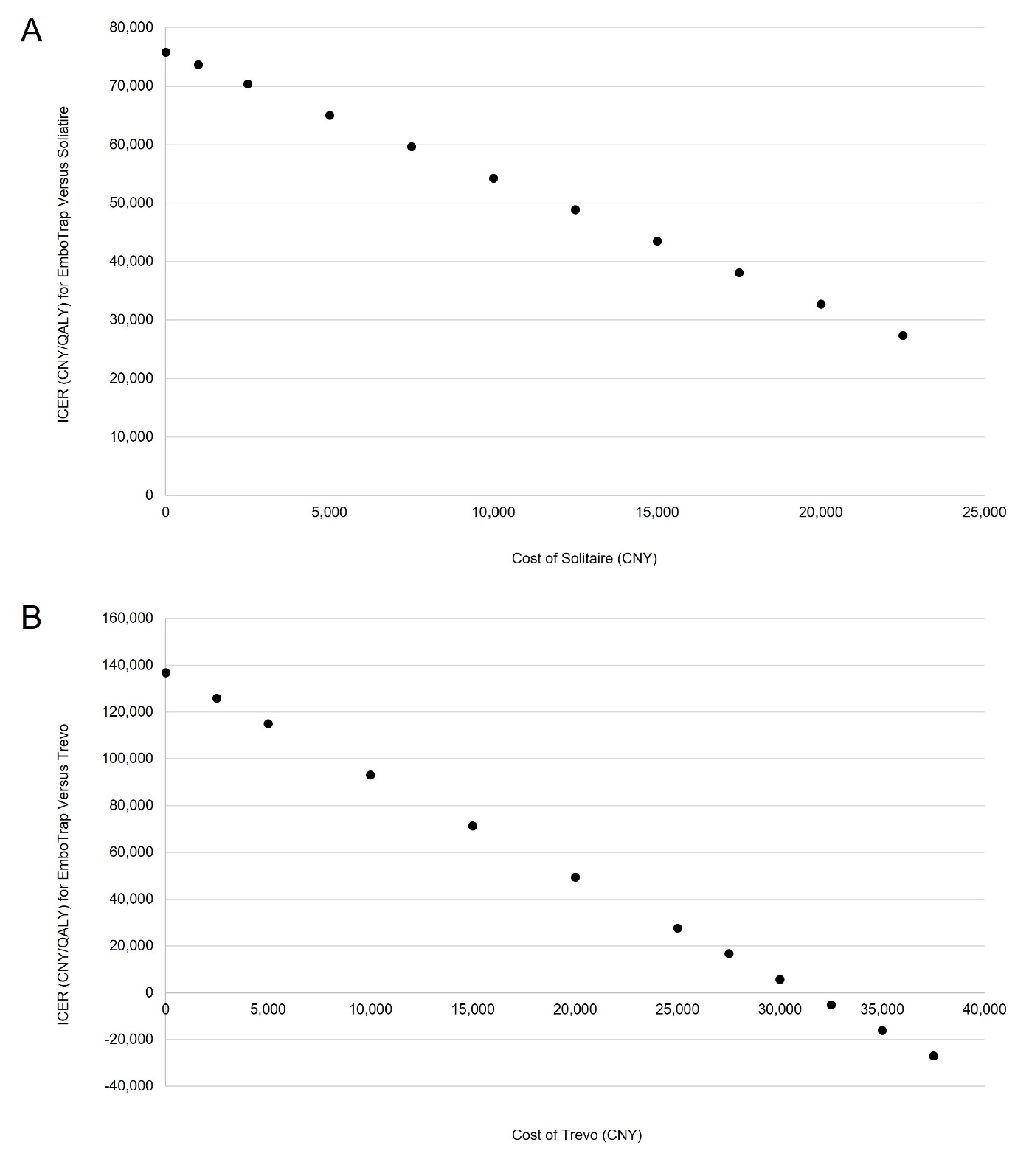


Note: To convert from 2022 CNY to 2022 United States Dollars ($), divide by 6.96 [37].

Abbreviations: CNY = Chinese Yuan; ICER = incremental cost-effectiveness ratio; QALY = quality-adjusted life-year.
